# Novel Back Propagation Optimization by Cuckoo Search Algorithm

**DOI:** 10.1155/2014/878262

**Published:** 2014-03-20

**Authors:** Jiao-hong Yi, Wei-hong Xu, Yuan-tao Chen

**Affiliations:** School of Computer and Communication Engineering, Changsha University of Science and Technology, Changsha, Hunan 410014, China

## Abstract

The traditional Back Propagation (BP) has some significant disadvantages, such as training too slowly, easiness to fall into local minima, and sensitivity of the initial weights and bias. In order to overcome these shortcomings, an improved BP network that is optimized by Cuckoo Search (CS), called CSBP, is proposed in this paper. In CSBP, CS is used to simultaneously optimize the initial weights and bias of BP network. Wine data is adopted to study the prediction performance of CSBP, and the proposed method is compared with the basic BP and the General Regression Neural Network (GRNN). Moreover, the parameter study of CSBP is conducted in order to make the CSBP implement in the best way.

## 1. Introduction

Though the traditional neural networks (such as BP) have been widely used in many areas, they have some inherent shortcomings. These disadvantages have become a major bottleneck that restricts their further development. In most cases, the gradient descent method is used in feedforward neural networks (FNN), which has the following main disadvantages.

1. Training slowly: many iterations are required in the gradient descent method in order to adjust weights and bias. Therefore, the training process takes long time.
2. It is easy to fall into local minimum, so that it cannot achieve the global minimum.
3. It is very sensitive to the choice of the initial weights and bias. Due to high influence on the performance for neural networks (NN), proper weights and bias must be carefully selected in order to obtain a more ideal network. If the selection of the weights and bias is improper, convergent speed of the algorithm will be very slow and the training process would take a long time.

Therefore, in order to enhance the performance of BP, many scholars are always striving for exploring a training algorithm that has a fast training speed, a global optimal solution, and a good generalization performance. Finding this training algorithm is also the main objective of the research in recent years.

Many metaheuristic methods have been proposed to solve optimization problems, such as the charged system search (CSS) [1], big bang-big crunch algorithm [2–5], harmony search (HS) [6–8], particle swarm optimization (PSO) [9–13], biogeography-based optimization (BBO) [14–18], firefly algorithm (FA) [19–23], differential evolution (DE) [24–27], krill herd (KH) [28–31], and bat algorithm (BA) [32–34].

In this paper, CS algorithm [35–37] that is a newly-developed metaheuristic method is used to optimize the weights and bias of BP. That is to say, CS is well capable of selecting the best initial weights and bias so as to construct the BP network instead of the randomly-generated weights and bias used in the basic BP. In order to prove the superiority of CSBP, it is used to solve the Italian wine classification problem. By comparing with the traditional BP and GRNN, this method has higher prediction accuracy and better generalization performance.

The remainder of this paper is organized as follows. The preliminaries including CS and BP are provided in Section 2.  Section 3 represents the detailed BP optimized by CS. Then, in Section 4, a series of comparison experiments on Italian wine classification problem are conducted. The final section provides our concluding remarks and points out our future work orientation.

## 2. Preliminary

### 2.1. CS Algorithm

CS method [35, 36] is a novel metaheuristic swarm intelligence [38] optimization method for solving optimization problems. It is based on the behavior of some cuckoo species in combination with the Lévy flights. In the case of CS, how far a cuckoo can move forwards in a step can be determined by the Lévy flights.

In order to describe CS algorithm more easily, Yang and Deb [35] idealized the behavior of the cuckoo species into the following three rules:

1. for all the cuckoos in the population, every one lays only one egg at a time and randomly selects a nest in order to place this egg;
2. the population cannot change the eggs with the best fitness in order to make the whole population evolve forward all the time;
3. the host bird discovers the cuckoo eggs with a probability *p*_*a*_ ∈ [0,1]. In this case, the cuckoo has no other choice and it has to build a fully new nest.

Based on the above hypothesis, the CS can be summarized as shown in Algorithm 1. We must point out that, for single objective problems, the cuckoos, eggs, and nests are equal to each other. So, we do not differentiate them in our works.

In order to make the balance of exploitation and exploration, CS uses a balanced combination of a local random walk and the global explorative random walk, controlled by a switching parameter *p*_*a*_. The exploitation step can be represented as

(1) $$\begin{array}{c} x_{i}^{t+1}=x_{i}^{t}+\beta s⨂H\left(p_{a}-\varepsilon \right)⨂\left(x_{j}^{t}-x_{k}^{t}\right), \end{array}$$

where *x*_*j*_^*t*^ and *x*_*k*_^*t*^ are two different randomly selected cuckoos, *H*(*u*) is a Heaviside function, *ε* is a random number, and *s* is the step size. On the other hand, the exploration step is implemented by using Lévy flights as follows:

(2) $$\begin{array}{c} x_{i}^{t+1}=x_{i}^{t}+\beta L\left(s,\lambda \right), \end{array}$$

where *L*(*s*, *λ*) = (*λ*Γ(*λ*)sin(*πλ*/2)/*π*)(1/*s* ^1+*λ*^), (*s*, *s*_0_ > 0),  *β* > 0 is the scaling factor, and its value can be determined by the problem of interest. More information of CS can be referred to in [39–41].

### 2.2. BP Network

BP network was proposed by a team of scientists led by Rumelhart and McCelland in 1986 which is an error back propagation algorithm according to the former train multilayer feedforward network. It is one of the most widely used neural network models. BP network can learn and remember a lot of input-output mapping model without prior mathematical equations that describe this mapping. The steepest descent method is used as the learning rules in order to adjust the weights and bias that can finally minimize the network error. In general, the topology of the BP network model includes input layer, hidden layer, and output layer. The number of layers and neurons in each hidden layer can be determined by the dimension of the input vector, and the output vector. In most cases, a single hidden layer is used in BP network.

BP network is a kind of supervised learning algorithm. Its main idea can be represented as follows. Firstly, training samples are input into the BP network, and then weights and bias are adjusted by using the error back propagation algorithm. This training process would minimize the error between the desired vector and the output vector. When the error is satisfied, weights and bias are remembered, which can be used to predict test samples. More information about BP can be referred to in [42].

## 3. CSBP

In the present work, the CS algorithm is used to optimize BP network. More specifically, the BP network is considered to be objective function (fitness function), and the weights and bias are optimized by the CS method in order to obtain the optimal weights and bias. The best weights and bias are well-suited to construct the BP that is significantly superior to the basic BP network.

**Figure 1:**
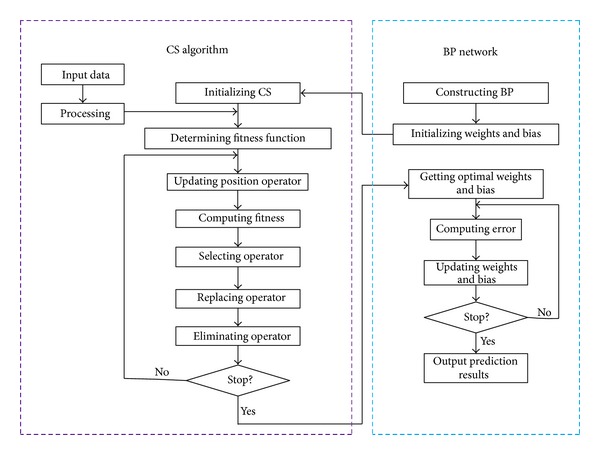
Flowchart of CSBP.

The process of the BP network optimized by the CS is divided into three parts: determining BP network structure, obtaining the best weights and bias through CS, and predicting through neural network. The structure of BP network in the first part is determined based on the number of input and output parameters, and then the length of each cuckoo individual in CS is determined accordingly. In the second part, CS method is applied to optimize the weights and bias of the BP network. Each individual in the cuckoo population includes all the weights and bias in BP, and it is evaluated by the fitness function. The CS method implements initializing CS, determining fitness function, updating position operator, selecting operator, replacing operator, and eliminating operator in order to find the cuckoo individual with the best fitness. This optimization process is repeated until the satisfactory weights and bias are found. In the last part, the BP network with the optimal weights and bias is constructed and is trained to predict the output. Based on the above analyses, the flowchart of the CSBP algorithm can be shown in Figure 1.

In CSBP, CS is applied to optimize the initial weights and bias of BP network, so that the optimized BP network has better predicted output. The elements in CSBP include initializing CS, determining fitness function, updating position operator, selecting operator, replacing operator, and eliminating operator in order to find the cuckoo individual with the best fitness. The detailed steps of the CS algorithm (see Figure 1) are as follows. 

(*1) Initializing CS.* Cuckoo individual is encoded in the real-coded form, and each individual is composed of real-number string that consists of the following four parts: connection weights between the hidden layer and output layer, connection weights between the hidden layer and the input layer, the bias in the output layer, and the hidden layer. Each cuckoo individual contains all the weights and bias in BP network. According to the weights and bias in BP network, a certain BP network can be constructed.

(*2) Determining Fitness Function*. The initial weights and bias of BP network can be determined according to the best individual. After training the BP network, it is used to predict the output. The fitness value of cuckoo individual *F* is the sum of the absolute error between the desired output and the predicted output *E* as follows:

(3) $$\begin{array}{c} F=k\left(\sum_{i=1}^{n}\left|y_{i}-o_{i}\right|\right), \end{array}$$

where *n* is the node number of the output layer in BP network and *k* is a coefficient. *y*_*i*_ and *o*_*i*_ are the desired output and the predicted output for the node *i* in BP network.

(*3) Updating Position Operator.* A cuckoo (say *i*) is randomly chosen in the cuckoo population and its position is updated according to (1). The fitness (*F*_*i*_) of the *i*th cuckoo at generation *t* and position *x*_*i*_(*t*) is evaluated by (3).

(*4) Selecting Operator*. Similarly, another cuckoo (say *j*, *i* ≠ *j*) is randomly chosen in the cuckoo population and its position fitness (*F*_*j*_) of the *i*th cuckoo at generation *t* and position *x*_*j*_(*t*) is evaluated by (3).

(*5) Replacing Operator*. If the fitness value of the cuckoo *i* is bigger than the cuckoo *j*, that is, *F*_*i*_ > *F*_*j*_, *x*_*j*_ is replaced by the new solution. 

(*6) Eliminating Operator.* In order to make the population in an optimum state all the time, ceil(*n*∗*p*_*a*_) worst cuckoos are removed in each generation. At the same time, in order to make the population size unchanged, ceil(*n*∗*p*_*a*_) cuckoos would randomly be generated. The cuckoos with the best fitness will be passed directly to the next generation. Here, ceil(*x*) rounds the elements of *x* to the nearest integers towards infinity.

*BP network* in CSBP (see Figure 1) is similar to an ordinary BP network, and the detailed steps can be represented as follows.

(*1) Determining BP Network Structure.* The weights and bias are randomly initialized, and then they are encoded according to the CS algorithm. The encoded weights and bias are input into the CS in order to optimize the BP network, followed by the CS algorithm (see Figure 1). 

(*2) Construct CSBP Network*. The optimal weights and bias obtained from the CS algorithm are used to construct CSBP network. The training set is used to train the network and the training error is calculated. When the training error meets the requirements, training of theCSBP network stops. 

(*3) Predicted Output*. The test set is input into the trained CSBP network to predict output.

## 4. Simulation

A classical wine classification problem (http://archive.ics.uci.edu/ml/datasets/wine) is used to test the prediction effectiveness of the CSBP network. Wine data that originated from UCI wine database records three different varieties of wine on the chemical composition analysis grown in the same region in Italy. Different kinds of wine are identified with 1, 2, and 3. Each sample contains 14 dimensions. The first dimension represents a class identifier, and the others represent the characteristics of wine. In these 178 samples, 1–59, 60–130, and 131–178 belong to the first, second, and third category, respectively. Each category is divided into two parts: training set and test set.

### 4.1. Comparisons of CSBP with BP and GRNN

In this section, CSBP is applied to solve wine classification problem, and the results are compared with the traditional feedforward neural networks (BP and GRNN).

For CSBP and BP, the neurons in input layer, hidden layer, and output layer are 13, 11, and 1, respectively. The length of encoded string number for each cuckoo individual is 166 that can be computed by the following equation: 13∗11 + 11 + 11∗1 + 1 = 166. That is, CS would find the minimum of a 166-dimension function.

**Figure 2:**
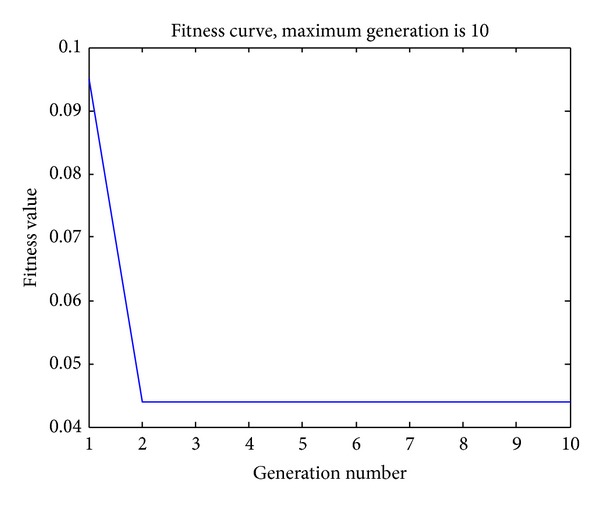
Convergent curve of CS with Max gen = 10,* Pop*size = 10, and *p*_*a*_ = 0.1.

Firstly, the performance of CS when optimizing the weights and bias is tested with discovery rate *p*_*a*_ = 0.1 and few population sizes (10) and maximum generations (10). The fitness curve can be shown in Figure 2. From Figure 2, it can be seen that fitness value sharply decreases from 0.095 to 0.045 within two generations. This means that CS can significantly minimize the training error, and it does succeed in optimizing the basic BP network.

In the next experiments, all the paraments are setted as follows. For BP network, epochs = 50, learning rate = 0.1, and objective = 0.00004. For GRNN, cyclic training method is used in order to select the best SPREAD value, making GRNN achieve the best prediction. For the CSBP, the BP network part has the same parameters with the basic BP; for CS algorithm part, we set discovery rate *p*_*a*_ = 0.1, population size NP = 50, and maximum generationMax gen = 50.

**Figure 3:**
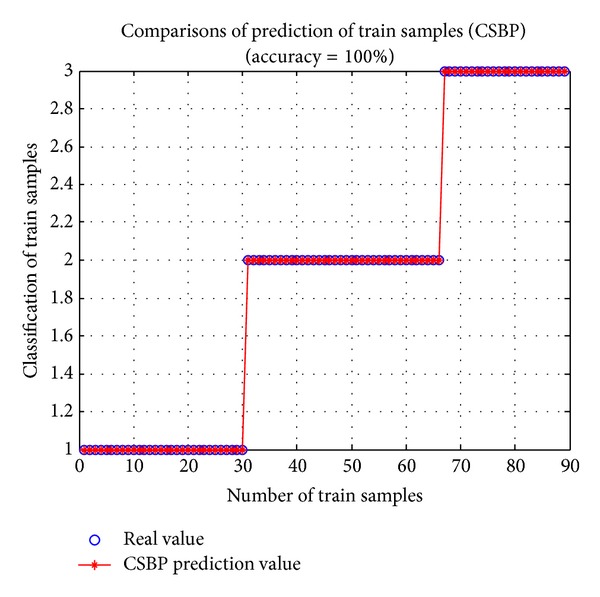
Prediction of train samples (CSBP) with Max gen = 50,* Pop*size = 50, and *p*_*a*_ = 0.1.

**Figure 4:**
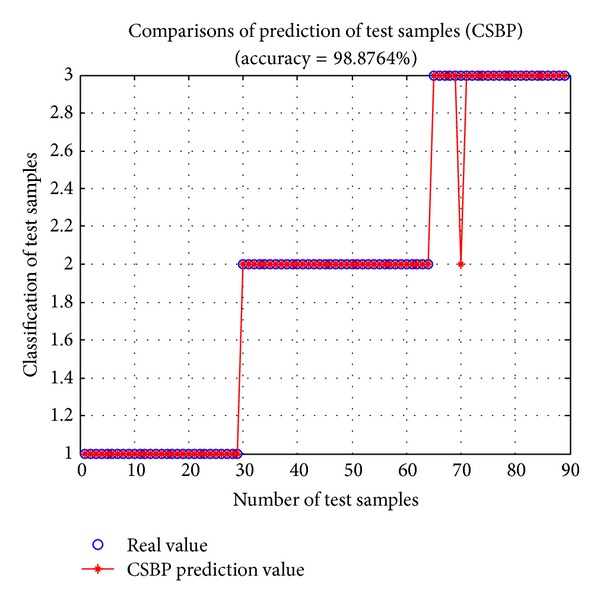
Prediction of test samples (CSBP) with Max gen = 50,* Pop*size = 50, and *p*_*a*_ = 0.1.

As intelligent algorithms always have some randomness, each run will generate different results. In order to get a typical statistical performance, 600 implementations are conducted for each method. The results are recorded in Figures 3 and 4 and Table 1.

From Table 1, for training set, the best performance and the average performance of BP, CSBP, and GRNN have little difference though CSBP performs slightly better than BP and GRNN. For the worst performance, CSBP is better than GRNN and is significantly superior to BP network. For test set, the overall prediction accuracy of CSBP is much better than BP and GRNN. In addition, the Std (standard deviation) of CSBP is clearly less than BP and GRNN. That is to say, CSBP would generate a more stable prediction output with little fluctuation. Moreover, from Figures 3 and 4, CSBP has a strong ability of solving the wine classification problem.

### 4.2. Parameter Study

As we are aware, parameter settings are of paramount importance to the performance of the metaheuristics. Here, the effectiveness of maximum generation, population size, and discovery rate will be analyzed and studied for CS algorithm.

#### 4.2.1. Influence of the Maximum Generation for CSBP

Firstly, the number of maximum generations (Max gen) is studied, and the results are shown in Table 2. Table 2 shows that, when Max gen is equal to 40, 50, or 100, CSBP can approach all training samples without error. However, prediction accuracy is not always getting better with the increment of maximum generation. From prediction accuracy of test set, it can be seen that, when the number of maximum generations increases from 10 to 100, the prediction accuracy of test set is gradually increased, decreased, and finally increased. Especially, when Max gen = 100, the prediction accuracy reaches maximum (89/89 = 100%). Look carefully at Table 2; it is observed that the prediction accuracy changes in a very small range. That is, CSBP is insensitive to the parameter Max gen. Meanwhile, though more generations (such as 100) have a perfect prediction accuracy, it would take a longer time in order to optimize the weights and bias. Taking into consideration all the factors we analyzed earlier, the maximum generation is set to 50 in our present work.

#### 4.2.2. Influence of the Population Size for CSBP

Subsequently, the influence of population size (NP) is studied (see Table 3). From Table 3, when NP is in the range [10, 100], especially equal to 100, CSBP can approach all training samples with little error. From prediction accuracy of test set, when the number of population size is equal to 100, the prediction accuracy of test set reaches maximum. Similar to the trend about Max gen, when the NP increases from 10 to 100, though prediction accuracy gradually increased, decreased, and finally increased, its fluctuation is little. This means that population size has little effect on the prediction accuracy of CSBP. In addition, when NP = 100, the prediction accuracy reaches maximum (89/89 = 100%). The prediction accuracy of NP = 50 is only inferior to NP = 100. However, NP = 50 would take shorter time and obtain relatively satisfactory results. Comprehensively considering, the population size is set to 50 in other experiments.

#### 4.2.3. Influence of the Discovery Rate for CSBP

Finally, in this section, the influence of the last important parameter discovery rate (*p*_*a*_) is studied through a series of experiments (see Table 4). From Table 4, it is clear that, when *p*_*a*_ is in the range [0, 1], the prediction accuracy of the CSBP is always bigger than 98.88% (88/89) for training samples. Due to CS, CSBP has a fast train speed. From the prediction accuracy of test set, when the discovery rate is equal to 0.1, the prediction accuracy of test set approaches the maximum 97.87% (87.1/89). Generally speaking, the prediction accuracy of CSBP for test samples varies a little with the incensement of the discovery rate. Comprehensively considering, the discovery rate *p*_*a*_ is set to 0.1 in our paper.

## 5. Conclusion

If BP network has bad initial weights and bias, it would fail to find the best solution. In order to overcome the disadvantages of BP, this paper uses the CS algorithm to optimize the weights and bias in the basic BP to solve the prediction problem. This method trains fast, can obtain the global optimal solution, and has good generalization performance. Most importantly, CSBP is insensitive to the initial weights and bias and the parameter settings of CS algorithm. We only need to input the training samples into the CSBP network, and then CSBP can obtain a unique optimal solution. By comparing to other traditional methods (such as BP and GRNN), this method has a faster and better generalization performance.

In future, our research highlights would be focused on in the following points. On the one hand, CSBP will be used to solve other regression and classification problems, and their results can be further compared to other methods, such as feedforward neural network [43], Wavelet Neural Network (WNN) [44, 45], and Extreme Learning Machine (ELM) [46, 47]. On the other hand, we will hybridize BP with some other metaheuristic algorithms, such as artificial plant optimization algorithm (APOA) [48], artificial physics optimization [49], flower pollination algorithm (FOA) [50], grey wolf optimizer (GSO) [51], and animal migration optimization (AMO) [52], so as to further improve the performance of BP.

## Figures and Tables

**Algorithm 1:**
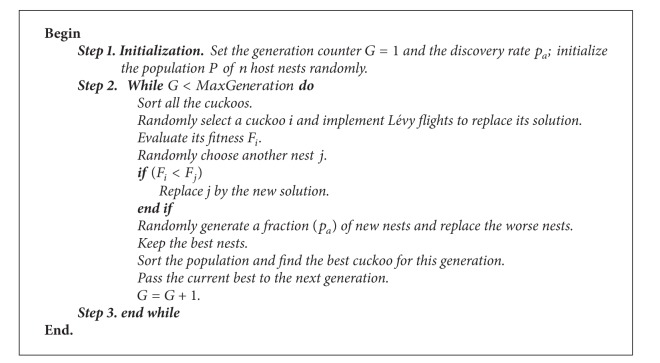
Cuckoo search.

**Table 1:** The accuracy of BP, CSBP, and GRNN.

	Train set				Test set			
	Mean	Best	Worst	Std	Mean	Best	Worst	Std
BP	87.08	**89**	52	2.67	84.12	**89**	43	3.80
CSBP	**88.78**	**89**	**88**	**0.44**	**87.33**	**89**	**82**	**0.97**
GRNN	88.50	**89**	84	0.74	85.58	**89**	79	1.34

**Table 2:** The accuracy of CSBP with different maximum generations.

Max gen	Train set				Test set			
	Mean	Best	Worst	Std	Mean	Best	Worst	Std
10	88.60	**89**	88	0.55	86.4	88	83	2.30
20	87.8	**89**	85	1.64	87.4	89	85	1.52
30	88	**89**	87	1.00	86.6	89	84	1.95
40	**89**	**89**	**89**	**0**	88.17	89	85	1.60
50	**89**	**89**	**89**	**0**	88.2	89	87	1.10
60	87.6	**89**	87	1.14	84.4	89	**77**	4.93
70	88	**89**	86	1.41	85	87	84	1.41
80	**89**	**89**	**89**	**0**	86.8	89	82	3.19
90	87.2	**89**	85	1.79	86.2	89	82	2.77
100	88.8	**89**	88	0.45	**89**	**89**	**89**	**0**

**Table 3:** The accuracy of CSBP with different population sizes.

NP	Train set				Test set			
	Mean	Best	Worst	Std	Mean	Best	Worst	Std
10	88.6	88	88	0.55	86.4	88	83	2.30
20	88.4	**89**	86	1.34	87.6	**89**	86	1.34
30	88.6	**89**	88	0.55	86.4	**89**	84	2.51
40	88.8	**89**	88	0.45	87.8	**89**	87	0.84
50	88.8	**89**	88	0.45	88.8	**89**	88	0.45
60	88.4	**89**	87	0.89	88.4	**89**	87	0.89
70	87.5	**89**	82	2.01	84.6	**89**	67	6.72
80	88.4	**89**	82	1.78	86.9	**89**	67	5.42
90	88.4	**89**	87	0.89	87.4	**89**	86	1.14
100	**89**	**89**	**89**	**0**	**89**	**89**	**89**	**0**

**Table 4:** The accuracy of CSBP with different discovery rates.

*p*_*a*_	Train set				Test set			
	Mean	Best	Worst	Std	Mean	Best	Worst	Std
0	88.4	**89**	**88**	0.52	84.7	**89**	77	3.30
0.1	88.6	**89**	87	0.70	**87.1**	**89**	**83**	**2.23**
0.2	88.2	**89**	87	1.03	86	**89**	77	3.56
0.3	88.4	**89**	86	1.08	84.9	**89**	75	5.17
0.4	**88.9**	**89**	88	**0.32**	85.9	**89**	76	4.45
0.5	88.1	**89**	83	1.91	84.6	**89**	76	4.92
0.6	88.4	**89**	86	1.08	86	**89**	81	3.01
0.7	88.5	**89**	87	0.71	86.4	**89**	82	2.50
0.8	88.2	**89**	86	1.14	85.7	**89**	77	3.56
0.9	88.1	**89**	83	1.91	86.9	**89**	82	2.51
1.0	88.1	**89**	85	1.29	85.8	**89**	76	4.15
